# Dextrose Administration and Resuscitation Outcomes in Patients with Blood Sugar Less Than 150 mg/dL during Cardiopulmonary Resuscitation: An Observational Data Analysis

**DOI:** 10.3390/jcm12020460

**Published:** 2023-01-06

**Authors:** Wachira Wongtanasarasin, Phichayut Phinyo

**Affiliations:** 1 Department of Emergency Medicine, Faculty of Medicine, Chiang Mai University, Chiang Mai 50200, Thailand; 2 Department of Emergency Medicine, UC Davis School of Medicine, Sacramento, CA 95817, USA; 3 Department of Family Medicine, Faculty of Medicine, Chiang Mai University, Chiang Mai 50200, Thailand; 4 Center for Clinical Epidemiology and Clinical Statistics, Faculty of Medicine, Chiang Mai University, Chiang Mai 50200, Thailand; 5 Musculoskeletal Science and Translational Research (MSTR), Chiang Mai University, Chiang Mai 50200, Thailand

**Keywords:** cardiac arrest, dextrose, low blood glucose, hypoglycemia, emergency department, survival

## Abstract

Low blood sugar is commonly found during cardiopulmonary resuscitation (CPR). However, current guidelines do not mention the importance of glucose testing and acute management for hypoglycemia during CPR. We intended to investigate the association between dextrose administration and resuscitation outcomes in patients with blood sugar less than 150 mg/dL during cardiac arrest in the emergency department (ED). We conducted a retrospective cohort study at a tertiary hospital between 2017 and 2020, including patients with intra-arrest blood glucose <150 mg/dL. Logistic regression with inverse probability treatment weighting (IPTW) was used. The primary outcome was the return of spontaneous circulation (ROSC). Secondary outcomes included survival to hospital admission and hospital discharge and favorable neurological outcomes at discharge. A total of 865 patients received CPR at the ED during the study period. Of these, 229 with low blood sugar were included (60 in the treatment group and 169 in the non-treatment group). The mean age was 59.5 ± 21.4 years. After IPTW, dextrose administration during CPR was not associated with ROSC (adjusted OR [aOR] 1.44, 95% CI 0.30–0.69), survival to hospital admission (aOR 1.27, 95% CI 0.54–3.00), survival to hospital discharge (aOR 0.68, 95% CI 0.20–2.29), and favorable neurological status (aOR 2.21, 95% CI 0.23–21.42). Our findings suggested that dextrose administration during CPR at the ED might not lead to better or worse resuscitation outcomes. Owing to the design limitations and residual confounding factors, strong recommendations for dextrose administration could not be formulated. Further evidence is needed from prospective trials to confirm the efficacy of dextrose during CPR.

## 1. Introduction

Cardiopulmonary arrest (CA) is acknowledged as one of the leading causes of death and disability worldwide. Each year, approximately 300,000 individuals in the United States are diagnosed with sudden cardiac arrest and receive cardiopulmonary resuscitation (CPR) in emergency departments (EDs) [1,2]. Currently, physicians and healthcare professionals who encounter patients with CA perform CPR following the American Heart Association (AHA) Guidelines for Advanced Cardiac Life Support (ACLS), which are updated every five years, the latest in 2020 [3].

Glucose is a vital resource that practically all cells and organisms utilize for a network of several metabolic processes [4]. Blood glucose (BG) is one of the important indicators to be measured during CA, as hypoglycemia was once mentioned in the previous guidelines as one of the reversible causes of arrest [5]. Since the 2010 ACLS Guidelines [6], however, hypoglycemia has been removed from the list of reversible causes of CA. In addition, the following edition did not discuss assessing BG and acute hypoglycemia therapy during CPR. A commonly used definition of hypoglycemia is a blood sugar level below 70 mg/dL (3.89 mmol/L) [7]. However, a cut-off level of hypoglycemia during CA was not definitely specified and varied greatly in the literature. In this study, low blood glucose was defined as a level below 150 mg/dL based on the findings of a previous study by Wang et al., which showed that CA patients with blood glucose levels below 150 mg/dL had poor neurological recovery [8]. Earlier evidence demonstrated that a hypoglycemic state during CA is associated with poor outcomes [8,9]. A recent case report, on the other hand, documented a rapid restoration of spontaneous circulation following dextrose treatment for hypoglycemia (10.8 mg/dL) [10]. Furthermore, a preclinical study illustrated that fatal ventricular arrhythmias occurred during severe hypoglycemia and could be diminished by intravenous dextrose infusion and prevented by the β-adrenergic blockade, suggesting that brain neuroglycopenia and the stimulating sympathoadrenal response mediated fatal cardiac arrhythmias during severe hypoglycemia [11]. However, a study by Wang et al. showed that the administration of dextrose during CPR was not associated with better outcomes, including survival to hospital discharge and good neurological status at discharge [9]. Currently, the recommendation for treating hypoglycemia during CA is still controversial. This study intended to investigate the association between dextrose administration and clinical outcomes in patients with blood sugar less than 150 mg/dL during CA at the ED.

## 2. Materials and Methods

### 2.1. Study Design and Setting

This therapeutic research was conducted with a retrospective observational cohort design at a single emergency department at Maharaj Nakorn Chiang Mai Hospital (MNCMH). MNCMH is a university tertiary hospital in northern Thailand that offers all specialties and 24 h emergency care by accredited emergency physicians. Our research was conducted in compliance with the Declaration of Helsinki statements. The institutional review board of the Faculty of Medicine, Chiang Mai University, approved the study protocol (Approval No. 7477/2020). This study was reported following the STrengthen the Reporting of OBservational studies in Epidemiology (STROBE) statement [12].

### 2.2. Participants

Patients who had a CA at MNCMH’s ED between January 2017 and December 2020 were eligible for this study. We included patients based on the following criteria: (1) age ≥18 years, (2) a documented lack of pulse requiring CPR in the ED, (3) no evidence or documentation of a do-not-attempt-resuscitation (DNAR) order before CA, (4) the measurement and documentation of their blood dextrose level during CPR, and (5) their blood glucose level was <150 mg/dL (8.3 mmol/L). A cut-off value of 150 mg/dL was chosen to define low blood glucose in this study since the previous study by Wang et al. demonstrated that cardiac arrest patients with intra-arrest BG levels of 150 mg/dl had poor neurological recovery [8].

Only the first episode of cardiac arrest was documented and included in this study if a patient’s cardiac arrest occurred more than once during an ED visit. Also, only the first sample was recorded if the blood glucose was obtained more than once in a single event. Patients who had an out-of-hospital cardiac arrest (OHCA) without prehospital ROSC and were transferred to the ED or who encountered cardiac arrest at the ED (EDCA) were included in this study. As there are multiple causes of cardiac arrest that need to be corrected and cardiac arrest patients are often administered with several agents, it is likely that dextrose administration might not be administered just for the correction of the blood glucose level. For instance, intravenous dextrose may be used together with calcium to correct hyperkalemia during CPR [13]. Thus, patients administered with dextrose and calcium might have a higher chance than those administered with glucose alone. For the other drugs (e.g., sodium bicarbonate, amiodarone, and adrenaline), we included them as confounders since there was no evidence regarding their use together with dextrose. In this study, we excluded patients who received calcium therapy during CA to decrease the amount of baseline confounding factors and alleviate the heterogeneity across the study groups since we believe that the decision to administer dextrose was biased by the intended judgment of the physician to treat hyperkalemia during resuscitation as recommended by the current AHA guidelines.

### 2.3. Data Collection and Outcome Measurements

We obtained the clinical data from our cardiac arrest registry. The abstractors in our study were paramedics and nurses with at least two years of experience working in the emergency department. Trained abstractors assessed participants’ hospital medical data while simultaneously entering them into the REDcap platform (Vanderbilt University, Nashville, TN, USA). We collected information on the patient’s age, sex, time of arrival, cardiac arrest mechanism, initial presenting rhythm, drugs delivered during cardiac arrest (adrenaline, amiodarone, lidocaine, dextrose, and bicarbonate), CPR duration, and the first intra-arrest BG acquired during CPR. Various point-of-care devices with varying accuracy and usability were used to measure the intra-arrest BG levels. In most cases, blood was obtained promptly after intravenous insertion and before any medication was administered during CPR.

The primary outcome was the return of spontaneous circulation (ROSC). Secondary outcomes included survival to hospital admission, survival to hospital discharge, and favorable neurological status at discharge, defined as having a cerebral performance category (CPC) score of 1 or 2. The CPC score was extracted retrospectively by reviewing the hospital records of each patient.

### 2.4. Statistical Analysis

We used the statistical data from the previous study, which provided a proportion of exposed subjects of 0.284 [8]; A study size of at least 210 patients was required to achieve a two-sided alpha error of 0.05 and 80% statistical power. All data were analyzed using Stata 16 (StataCorp, College Station, TX, USA). The categorical variables were presented using numbers and percentages and analyzed using chi-squared tests. The continuous variables were presented using means and standard deviations and analyzed using student *t*-test statistics. A two-tailed *p*-value of 0.05 was used to establish statistical significance. The odds ratio (OR) was selected as the measurement endpoint. Standardized differences (STD) were employed to compare the clinical features of the two groups. An STD score of 10% or higher in either direction was considered significant and may indicate potential confounding by indication, or confounding by contraindication, at baseline [14]. We used inverse probability treatment weighting (IPTW) to adjust the imbalance of prognostic factors [15]. Firstly, we developed a multivariable logistic regression model to predict the likelihood of being administered dextrose during CPR. The propensity model contained the following elements: sex, age, time of cardiac arrest, mechanism of cardiac arrest, location of cardiac arrest, initial shockable rhythm, drugs administered during CPR, including adrenaline, amiodarone, lidocaine, and bicarbonate, total CPR duration, and the intra-arrest blood glucose level. The model prognosticated that the probabilities of being administered dextrose were used as treatment weights for the dextrose administration group. In contrast, the model predicted probabilities of not being administered with dextrose during CPR were used as treatment weights for another group without dextrose administration. To decrease the variability of the estimated treatment effect owing to extremely large weights on both ends, we performed a weight truncation at the 1st percentile and the 99th percentile [15]. We developed a balanced diagnostic plot to assess the difference in characteristics between the weighted and unweighted samples. The features with an absolute STD of more than 0.10 after accounting would be adjusted in the weighted logistic regression model for double robustness [15,16]. A weighted multivariable logistic regression was performed to assess the association between dextrose administration during CPR and the resuscitation outcomes. We analyzed the regression model separately for each endpoint.

Moreover, we performed an additional subgroup analysis according to the location of cardiac arrest (OHCA and IHCA) as recommended by the Utstein reporting guidelines [17]. We created an extra model using multivariable logistic regression analysis to predict the possibility of being prescribed dextrose, one model for the OHCA patients and another model for the cardiac arrest patients at the ED. Noticeably, the location of cardiac arrest was not included in these models. Accordingly, an additional subgroup analysis was also performed, stratified by the presumed mechanism of cardiac arrest: traumatic and non-traumatic mechanisms, because these two mechanisms had different prognoses and outcomes [18,19].

## 3. Results

### 3.1. Patient Characteristics

**Figure 1:**
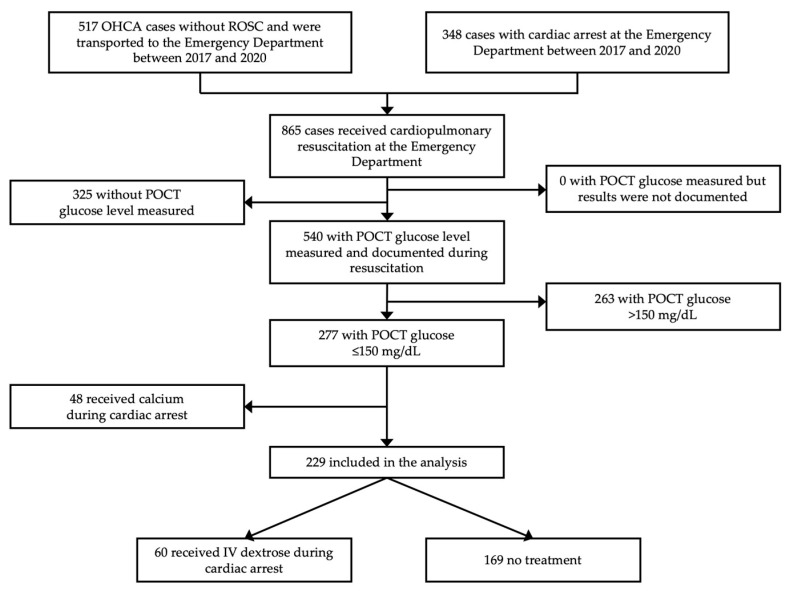
Study flow diagram of the study cohort. IV, intravenous; OHCA, out-of-hospital cardiac arrest; POCT, point-of-care testing; ROSC, return of spontaneous circulation.

A total of 865 cardiac arrest events were recorded during the study period. After excluding the patients without intra-arrest blood glucose measurements, patients with an intra-arrest blood glucose of more than 150 mg/dL, and patients who received calcium therapy during CPR, 229 remaining patients were included in this study (Figure 1). The average age was 59.5 years, and the majority were male (62.9%). In this study, the overall percentages of ROSC, survival to hospital admission, survival to hospital discharge, and favorable neurological status rates were 55.5%, 35.4%, 13.5%, and 5.5%, respectively. Except for the time, location of cardiac arrest, and lidocaine administration, the remaining baseline parameters differed across the two groups (Table 1). The intra-arrest blood glucose level was the most notable difference between the two groups (STD +3.382). Most of the mechanisms of cardiac arrest were non-trauma (78.2%) and out-of-hospital (69.6%). The treatment group had a longer total CPR duration (STD +0.195), whereas the initial shockable rhythm was higher in the non-treatment group (STD −0.261).

### 3.2. Outcomes

**Figure 2:**
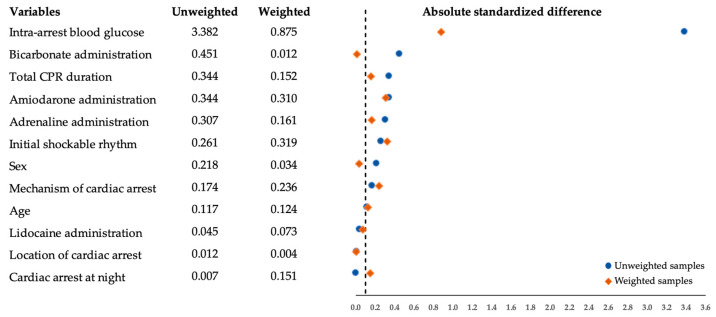
Balance in prognostic factors at baseline based on absolute standardized differences between the unweighted and weighted samples. CPR, cardiopulmonary resuscitation.

The absolute STD between the unweighted and weighted samples is illustrated in Figure 2. Almost all variables were unbalanced before matching. We performed a weight truncation at the 1st and 99th percentiles. At this point, three patients with extreme weight were excluded, leaving 226 patients for analysis. Several components remained imbalanced (absolute STDs >0.1) after adjustment with the IPTW. All these unbalanced factors, however, were included in the weighted multivariable logistic regression analysis. Table 2 demonstrates the association between each clinical endpoint and dextrose administration during the cardiac arrest of the study population.

**Figure 3:**
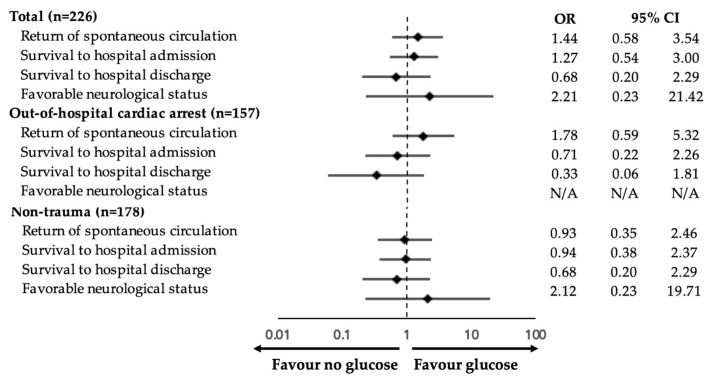
Weighted multivariable logistic regression analysis of each outcome in three different models, stratified by the location and mechanism of cardiac arrest. CI, confidence interval; OR, odds ratio.

Figure 3 depicts the odds ratio for each outcome grouped by the location and mechanism of cardiac arrest. Dextrose administration during CPR was not associated with improved ROSC (OR 1.44, 95% CI 0.58–3.54, *p* = 0.43), the rate of survival to hospital admission (OR 1.27, 95% CI 0.54–3.00, *p* = 0.59), the rate of survival to hospital discharge (OR 0.68, 95% CI 0.20–2.29, *p* = 0.53), and favorable neurological status (OR 2.21, 95% CI 0.23–21.42, *p* = 0.49). For selected groups, administering dextrose to OHCA and non-traumatic patients during CPR was not associated with better chances of resuscitation outcomes (Figure 3).

## 4. Discussion

In this study, we discovered that dextrose administration during CPR in patients with low blood glucose (<150 mg/dL) might not result in additional benefits or harmful effects.

In the 2005 AHA ACLS algorithm, hypoglycemia was listed as one of the reversible causes and probable contributing factors of cardiac arrest [5]. However, no evidence was provided to support its recommendation. Subsequently, the AHA removed hypoglycemia from the reversible causes of CA (the mnemonic “5 H’s and T’s”) in the following ACLS algorithm with no evidence [6]. To clarify, our institutional protocol applied the AHA ACLS algorithm, which did not include routine checking for hypoglycemia and delivering dextrose. As a result, when ED personnel realized the patient had gone into cardiac arrest, they immediately initiated CPR, defibrillation when indicated, intravenous access, and airway management. Although no explanation was stated for the removal of hypoglycemia from the “H’s and T’s” in the algorithm, one hypothesis was that it pushed physicians to administer dextrose to all patients in cardiac arrest. Expected dextrose delivery in cardiac arrest is not advised since it is linked with increased neurologic morbidity and a worse likelihood of survival to hospital discharge [20]. Nonetheless, our previous study found that intra-arrest blood glucose levels less than 100 mg/dL were associated with a lower chance of sustained ROSC [9]. However, according to this study, administering dextrose might not increase the chance of ROSC. Furthermore, hyperglycemia after ROSC was also related to prolonged recovery durations and worse neurological results, regardless of whether dextrose was administered [21,22,23,24]. The mechanism behind this is still debatable. Several studies have documented that hyperglycemia during times of cerebral ischemia stimulates anaerobic glycolysis, increasing lactate generation and intracellular acidosis [25,26]. 

On the other hand, the practice of routine dextrose administration in patients with suspected hypoglycemia-related events, such as seizures, altered mental status, weakness, or even cardiac arrest, was based not only on concerns that untimely treatment may result in irreversible brain damage but also on the inappropriate assumption that dextrose delivery was safe to patients who were neither normoglycemic nor hyperglycemic [20]. Empirical therapy with dextrose has long been suggested for any patient presenting with a coma of unknown etiology [27]. Nonetheless, strong evidence cast doubt on this practice [28]. Peng et al., using data from the large get with the guidelines-resuscitation (GWTG-R), found that dextrose was delivered to 4% of all IHCA patients [20]. Dextrose treatment was shown to be inversely related to favorable outcomes and survival in both multivariable regression and propensity-matched analyses [20]. However, this study did not account for the intra-arrest blood glucose level. Notably, since the degree of hypoglycemia was one of the indications for administering dextrose, the effect of dextrose administration may be influenced by confounding by indications, leading to exaggerated correlations without correcting for hypoglycemia or blood glucose levels. As a result, our study included the intra-arrest blood glucose level in the IPTW model for predicting the chance of receiving dextrose during CPR.

Overall, our current study contributes to the growing body of literature regarding the association between dextrose administration during CPR and resuscitation outcomes. Although earlier studies documented that a low intra-arrest blood glucose level was strongly correlated with poor outcomes [8,9], this study highlights that dextrose might not provide a significant additional benefit or harmful effect. The correction of hypoglycemia during cardiac arrest might not result in a better outcome, questioning the importance of dextrose administration during CPR. We proposed two possible explanations for this phenomenon. First, in a rat model, it was demonstrated that severe hypoglycemia caused a variety of fatal arrhythmias, including premature ventricular contraction, ventricular tachycardia, and complete bundle branch block. When beta-adrenergic blockers were provided, cardiac arrhythmias subsided, and hypoglycemia-related events were eliminated, suggesting that an excessive sympathoadrenal pathway might partially account for hypoglycemia-related death [11]. Second, low blood sugar during CPR may simply be one of the side effects of serious illness. Researchers of the NICE-SUGAR trial discovered that after correcting for the confounding effects of the baseline characteristics and post-randomization confounders, the link between hypoglycemia and mortality was mitigated [29]. Interestingly, they also observed that for patients not being treated with insulin, the link between hypoglycemia and mortality was increased, and the time to death was shorter compared to those receiving insulin. They proposed these findings: “spontaneous hypoglycemia”, or hypoglycemia not triggered by insulin administration and its significant relationship with poor outcomes, strongly suggested that hypoglycemia may simply be a disease indicator, and hence hypoglycemia-targeted treatments may be unsuccessful. The findings of our study should, however, be considered hypothesis-generating, and more evidence from larger studies with experimental designs is still highly encouraged. We hypothesize that dextrose may contribute some advantages during resuscitation because it normalized the intra-arrest blood glucose levels, which resulted in a better chance of ROSC and increased survival. For example, a large, randomized-controlled trial investigating the efficacy of dextrose during CPR is warranted.

Several limitations need to be considered in this study. Firstly, our study was based on a highly chosen group, with only 32.0% (277/865) of all screened patients enrolling. Most patients were removed due to a lack of intra-arrest measurement (37.6%, 325/865), which was most likely caused by the removal of hypoglycemia from the list of reversible causes of cardiac arrest in the resuscitation recommendations. The glucose measurement during CPR depends on each physician’s judgment since the current guidelines do not mention this procedure. In addition, patients who received calcium were removed since we hypothesized that these patients might receive dextrose as one of the treatments for treating hyperkalemia during CA, as recommended by the current AHA guidelines [3]. Furthermore, we did not collect the exact reason why these medications were administered during cardiac arrest. A further, well-controlled trial could handle this issue. Secondly, the study data were observational, which could only demonstrate an association, not a causal link, between the independent and dependent factors. This study could not investigate the exact explanations behind the association between dextrose administration and outcomes. This study, nevertheless, applied a cut-off value of 150 mg/dL from the previous literature to represent a “low blood sugar” status, resulting in some controversies in clinical practice since there is no consensus value for hypoglycemia during CPR. Furthermore, despite applying multivariable analysis to compensate for the impacts of measured confounding variables, the effects of unmeasured and residual confounding factors may still distort the results. Although we attempted to minimize biases by applying the IPTW method, the STD for the intra-arrest blood glucose variable after weighting was still high (an absolute STD of 0.875), causing an impact on the likelihood of patients receiving dextrose. A randomized trial is warranted to solve this issue through further study. Pre-arrest events that resulted in relatively low blood glucose levels, pre-arrest medication statuses for diabetes management, past medical histories of diet and nutritional statuses, and the reasons why intra-arrest blood glucose levels were tested, for example, might all be relevant confounding variables that could not be collected and adjusted during the analysis. A prospective, well-designed clinical trial is required to address these issues.

## 5. Conclusions

Dextrose administration to patients with low blood sugar levels during cardiac arrest in the emergency department was unlikely to provide any additional benefit or harm. Even though strong recommendations could not be concluded due to limitations in terms of the design and residual confounding variables, we proposed that blood glucose testing during CPR is considered reasonable. Further large-scale prospective research focusing on the efficacy of dextrose during CPR is still required.

## Figures and Tables

**Table 1:** Baseline characteristics, clinical features, and interventions of the study cohort.

Variables	All Patients (*n* = 229)	Treatment Group (*n* = 60)	Non-Treatment Group (*n* = 169)	STD
Age (years), mean ± SD	59.5 ± 21.4	62.0 ± 20.7	58.7 ± 21.7	+0.157
Age over 65, *n* (%)	97 (42.4)	28 (46.7)	69 (40.8)	+0.117
Male, *n* (%)	144 (62.9)	33 (55.0)	111 (65.7)	−0.218
Time of cardiac arrest, *n* (%)				−0.006
8.01–16.00	94 (41.1)	25 (41.7)	69 (40.8)	
16.01–24.00	63 (27.5)	16 (26.7)	47 (27.8)	
24.01–8.00	72 (31.4)	19 (31.7)	53 (31.4)	
Arrest at night, *n* (%)	72 (31.4)	19 (31.7)	53 (31.4)	+0.007
Location of cardiac arrest, *n* (%)				+0.045
Out-of-hospital	160 (69.9)	41 (68.3)	119 (70.4)	
Emergency department	69 (30.1)	19 (31.7)	50 (29.6)	
Traumatic mechanism, *n* (%)	50 (21.8)	10 (16.7)	40 (23.7)	−0.174
Initial shockable rhythm, *n* (%)	43 (18.8)	7 (11.7)	36 (21.3)	−0.261
Medications administered during cardiac arrest, *n* (%)				
Adrenaline	214 (93.5)	59 (98.3)	155 (91.7)	+0.307
Amiodarone	17 (7.4)	1 (1.7)	16 (9.5)	−0.344
Lidocaine	8 (3.5)	2 (3.3)	6 (3.6)	−0.012
Bicarbonate	78 (34.1)	30 (50.0)	48 (28.4)	+0.451
CPR duration (minutes), mean ± SD	18.4 ± 13.2	20.2 ± 12.2	17.7 ± 13.5	+0.195
CPR duration, *n* (%)				+0.344
<5 min	39 (17.0)	5 (8.3)	34 (20.1)	
5–15 min	74 (32.3)	18 (30.0)	56 (33.1)	
15–30 min	82 (35.8)	26 (43.3)	56 (33.1)	
>30 min	34 (14.9)	11 (18.3)	23 (13.6)	
Intra-arrest blood glucose level (mg/dL), mean ± SD	91.8 ± 40.4	42.3 ± 20.2	109.3 ± 29.9	−2.617
Intra-arrest blood glucose level <70 mg/dL, *n* (%)	72 (31.4)	57 (95.0)	15 (8.9)	+3.382

CPR, cardiopulmonary resuscitation; SD, standard deviation; STD, standardized difference.

**Table 2:** Clinical outcomes for receiving intravenous dextrose during cardiac arrest.

Outcomes	Unadjusted Odds Ratios (95% CIs)	*p*-Value	Adjusted Odds Ratio * (95% CIs)	*p*-Value
Return of spontaneous circulation	0.51 (0.28–0.93)	0.03	1.44 (0.58–3.54)	0.43
Survival to hospital admission	0.72 (0.38–1.36)	0.31	1.27 (0.54–3.00)	0.59
Survival to hospital discharge	0.54 (0.20–1.48)	0.23	0.68 (0.20–2.29)	0.53
Favorable neurological outcome at hospital discharge	0.55 (0.11–2.60)	0.29	2.21 (0.23–21.42)	0.49

* Double adjustment in the weighted samples with age, arrest mechanism, initial shockable rhythm, total cardiopulmonary resuscitation duration, intra-arrest blood glucose, time of arrest, and medications administered during cardiac arrest, including adrenaline and amiodarone. CI, confidence interval.

## Data Availability

Not applicable.
